# Subjective Trusts for the Control of Mobile Robots under Uncertainty

**DOI:** 10.3390/e24060790

**Published:** 2022-06-05

**Authors:** Eugene Kagan, Alexander Rybalov

**Affiliations:** 1 Department Industrial Engineering, Ariel University, Ariel 4076414, Israel; 2 Laboratory for AI, Machine Learning, Business and Data Analytics, Tel Aviv University, Tel Aviv 6997801, Israel; alxndr_r@yahoo.com

**Keywords:** subjective trust, mobile robot, mobile neuron, swarm, control

## Abstract

The paper deals with the methods of handling imperfect information and the control of mobile robots acting under uncertainty. It is assumed that the robots act in autonomous regime and are controlled internally without referring the externally defined probabilities of the states and actions. To control the activity of the robots, we suggest the novel multi-valued logic techniques based on the recently developed measures, known as the subjective trusts. In addition to specification of the robots’ movements, such a technique allows for direct definition of the robots’ swarming using the methods of artificial neural networks with mobile neurons. The suggested methods are verified by numerical simulations and running examples. The resulting framework forms a basis for processing non-probabilistic uncertainties and making individual decisions with imperfect information.

## 1. Introduction

Functionality of autonomous systems includes three main processes: observing and measuring the environmental and system’s states, making decisions and prescribing the future actions, and conducting the chosen action aimed to change the state of the environment or of the system. In the case of complete certainty in observations and errorless decision making and acting, the models of control implement the methods originated by Maxwell in 1868 [1]. Following this approach, behavior of the system is defined by a certain dynamical system that prescribes the further actions with respect to the current observations. In the case of uncertainty, the control process is more complicated and requires handling imperfect or incomplete information about the states of the environment and of the system, processing inaccurate and ambiguous decisions and correcting erroneous actions [2,3].

Starting in the later 1950s, operating with uncertainties in the autonomous systems control is based on probabilistic methods [4,5,6]. It is assumed that the uncertainties in the observation results and the errors in the decisions and actions can be defined in the terms of random variables and their probabilities.

However, the use of probabilistic methods involves presumptions, which follows from the nature of probability, but do not hold in the framework of autonomous systems control. The difficulties in application of the probabilistic methods in the control of the swarm are due to two reasons.

The first is inconsistence of the probabilities to the considered situation. For example, consider a driver in the traffic flow who observes a pedestrian at the roadside. The probability that the pedestrian will cross the road on the red light is close to zero, but since it is possible, each experienced driver is ready to brake near the crosswalk. For the other example, consider observation of unknown or incompletely defined objects in a fog. Since the objects are unknown, the distances to the objects in the fog are also undefined. Hence, the probability that the certain object is observed is undefinable. Most such difficulties are considered in detail in the possibility theory [7,8].

The second reason is more complicated and follows from the nature of decision-making in the swarms, which does not imply direct minimization of the probabilities of outcomes or maximization of the probabilities of rewards (see the classical results by Kahneman and Tversky [9] and their recent validation [10]). Even in the simple case of motion toward the single target, an optimal behavior of the swarm involves a certain level of altruism of the swarm members such that in some steps the agents deny their movements to allow the movements of their neighbors [11]. In each specific task, such steps can be specified or at least approximated by stochastic optimization, however, in the general case, control of the swarm and the reactive activity of its members that leads to near optimal teleological behavior is still unclear.

In the paper we address a novel method for handling uncertainty based on the recently developed subjective trust measures [12,13]. Together with the parameterized version of the uninorm [14] and absorbing norm [15] aggregators, these measures form a formal algebra [16,17], which, in a certain sense, connects probabilistic and possibilistic approaches. Recently these measures were applied for formal description of prospects and demonstrated good correspondence with the experimental results [12]. In this paper, we continue this direction and define control of the swarm that allows to count the altruistic behavior of the agents without considering specific models and optimization of the swarm activity.

Starting from the observed relation between the subjective trusts, possibilities and necessities, we define the extended version of the uninorm and absorbing norm aggregators and the extended version of the algebra with these aggregators. The obtained algebra is considered as the algebra of control variables, which are governed directly by the decisions made by the means of the same algebra. Such an approach allows resolving uncertainties in the decision making and in the control at the same stage of the system’s evolution using the same tools. In addition, since the suggested algebra acts on the bounded interval, the processes are naturally limited, which prevents divergence of the controls even in the cases of erroneous decisions.

The suggested method is illustrated by the example of control of mobile robots. In the example, we start from the known model of a neural network with mobile neurons [18] described using subjective trusts [13] and extend this model to the use of control variables in the defined algebra. Then, swarming of mobile robots [19,20] is defined by direct implementation of the methods widely used in the descriptions of the ensembles of the neurons [21]. However, in contrast to the known methods, mobility of the neurons in the network and, consequently, of the robots in the swarm, is based both on the internal states of the neurons and the robots and on the states of the synapses, which are the values of the interconnections. In addition, we define the process of separation and unification of the synapses and specify the corresponding motion of the neurons and robots.

It should be noticed that the first attempt to use the concept of trusts for control of the mobile robots most likely appeared in the works [22,23]. In this performance centric method, the robot’s behavior is defined by the human-to-robot trust and by the human self-confidence. In other words, the higher the trust to the robot, the higher the quality of its performance and, similarly, the trust to the human is higher the better the human controls the robot. The suggested method, in contrast, can be considered as decision centric, in which the trust is governed by a priori defined algebraic rules, and the performance of the robot is a result of the operations with the trusts.

In addition, in the suggested control method the robot is defined as a dipole consisting of two neurons. However, in contrast to the original dipole activity [24], in the suggested model the dipoles are also governed by operators of the suggested algebra.

Numerical simulations verify the methods and demonstrate their convenience for definition of internal control under uncertainty with imperfect information about the states of the system and its actions. Computational complexity of the algorithms of robots’ control depends on the implemented methods of calculation of the subjective trusts. In the simulations, the trusts were calculated using hyperbolic tangent function, which does not add complexity to the algorithms.

## 2. Algebra of Control Variables

Control of the mobile robot is defined in the extended version of recently developed algebra that allows consideration in the same framework for both logical and arithmetical operations. We start with general definition of this algebra of multivalued logic and then present its extension on the control variables.

### 2.1. Algebraic Structure for Multivalued Logic

Let ${⊕}_{\theta }:\left[0,\;1\right]\times \left[0,\;1\right]\to \left[0,\;1\right]$ be a uninorm (or uninorm aggregator) [14] with neutral or identity element θ∈[0, 1], and ${⊗}_{\vartheta }:\left[0,\;1\right]\times \left[0,\;1\right]\to \left[0,\;1\right]$ be an absorbing norm (or absorbing norm aggregator also known as null norm) [15] with absorbing element ϑ∈[0, 1]. The first aggregator, with respect to the value of θ, extends Boolean and and or operators and the second aggregator extends Boolean not xor operator.

Similar to the Boolean binary operators, the uninorm ${⊕}_{\theta }$ and absorbing norm ${⊗}_{\vartheta }$ are symmetric and meet the commutative and associative properties. In addition, the uninorm ${⊕}_{\theta }$ is transitive. The neutral θ and absorbing ϑ elements play the role of unit and zero for their operators, respectively, such that for any x∈[0, 1] it holds that $\theta {⊕}_{\theta }x=x$ and $\vartheta {⊗}_{\vartheta }x=\vartheta$. It was proven [25] that for any x,y∈[0, 1] there exist the functions u:(0, 1)→(−∞,∞) and v:(0, 1)→(−∞,∞) called generator functions such that

(1) $$x{⊕}_{\theta }y=u^{-1}\left(u\left(x\right)+u\left(y\right)\right),$$

(2) $$x{⊗}_{\vartheta }y=v^{-1}\left(v\left(x\right)\times v\left(y\right)\right).$$

Generator functions u and v are continuous, strictly monotonously increasing with the zeroes u(θ)=v(ϑ)=0 and with the limits $\underset{x\to 0}{\lim}u\left(x\right)=\underset{x\to 0}{\lim}v\left(x\right)=-\infty$ and $\underset{x\to 1}{\lim}u\left(x\right)=\underset{x\to 1}{\lim}v\left(x\right)=+\infty$. The last property allows definition of the uninorm and absorbing norm on the boundary values 0 and 1 of the interval [0, 1].

It is easy to show [17] that the inverse functions $u^{-1}:\left(-\infty ,\infty \right)\to \left(0,\;1\right)$ and $v^{-1}:\left(-\infty ,\infty \right)\to \left(0,\;1\right)$ have the same properties as probability distributions and, in general, can be defined by any positive sigmoid functions with the values in the interval [0, 1]. Then, generator functions u and v are the inverses of the corresponding sigmoid functions and can be considered as quantile functions.

The values from the interval [0, 1] together with the operations defined by the uninorm and the absorbing norm form an algebra $A=\left(\left[0,1\right],{⊕}_{\theta },{⊗}_{\vartheta }\right)$, in which uninorm ${⊕}_{\theta }$ acts as a summation operator with zero θ and absorbing norm ${⊗}_{\vartheta }$ acts as a multiplication operator with the zero ϑ [16,17]. For completeness, algebra A also defined the operations

(3) $$x{⊖}_{\theta }y=u^{-1}\left(u\left(x\right)-u\left(y\right)\right),$$

(4) $$x{⊘}_{\vartheta }y=v^{-1}\left(v\left(x\right)/v\left(y\right)\right),$$

where v(y)≠0.

Algebra A extends the Boolean algebra B= 〈{0, 1}, ∧, ∨〉, which with usual binary conjunction ∧ and disjunction ∨ operators, and its multivalued version ℬ= 〈[0, 1], ⋏, ⋎〉 defined using t-norm and t-conorm [26]. In addition, algebra A can be considered as an arithmetic system on the interval [0, 1] in which uninorm ${⊕}_{\theta }$ and absorbing norm ${⊗}_{\vartheta }$ are associated respectively with the weighted arithmetical summation “+” and multiplication “×”. Consequently, operations ${⊖}_{\theta }$ and ${⊘}_{\vartheta }$ are associated, respectively, with the weighted arithmetical subtraction “−” and division “/”.

In general, algebra A is non-distributive, that is

(5) $$\left(x{⊕}_{\theta }y\right){⊗}_{\vartheta }z\neq \left(x{⊗}_{\vartheta }z\right){⊕}_{\theta }\left(y{⊗}_{\vartheta }z\right),$$

while u≠v or θ≠ϑ; however, if u=v or θ=ϑ then the distributivity property holds [16,17].

Assume that generator functions u and v are equivalent with equivalent neutral and absorbing elements and denote w=u=v and η=θ=ϑ. For an event A the trusts τ(A) about A are interpreted as follows:

1. τ(A)=1 means that A is necessary and τ(A)=0 means that A is impossible;
2. $\tau \left(A\right)=w^{-1}\left(1\right)$ means that A is probable and $\tau \left(A\right)=w^{-1}\left(-1\right)$ means that A is improbable;
3. $\tau \left(A\right)=w^{-1}\left(w^{-1}\left(1\right)\right)$ means that A is possible and $\tau \left(A\right)=w^{-1}\left(-w^{-1}\left(1\right)\right)$ means that A is unnecessary.

Such interpretation means that if, for example, the trust in the event A is $\tau \left(A\right)=w^{-1}\left(1\right)$, then the probability of the event A is p(A)=1 and vice versa, and if the trust in the event A is $\tau \left(A\right)=w^{-1}\left(-1\right)$, then the probability of the event A is p(A)=0 and vice versa. For the possibility and the necessity, the interpretation is similar.

For example, assume that generator function w is

(6) $$w\left(x\right)=\ln\left(x^{b}/\left(1-x^{b}\right)\right),\;\;\;x\in \left(0,\;1\right),$$

where $b=-1/\log_{2}\left(\eta \right)$. Then the inverse $w^{-1}$ of the generator function is a sigmoid function

(7) $$w^{-1}\left(\xi \right)={\left(\exp\left(\xi \right)/\left(1+\exp\left(\xi \right)\right)\right)}^{1/b},\;\;\;\xi \in \left(-\infty ,\infty \right).$$

**Figure 1:**
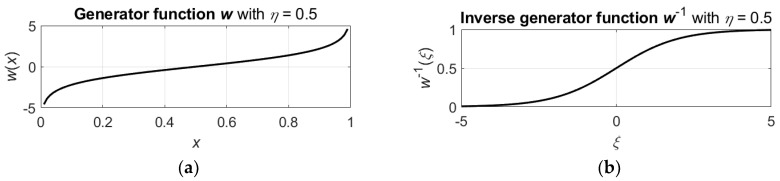
Generator function (**a**) and inverse generator function (**b**).

These functions are shown in Figure 1.

**Figure 2:**
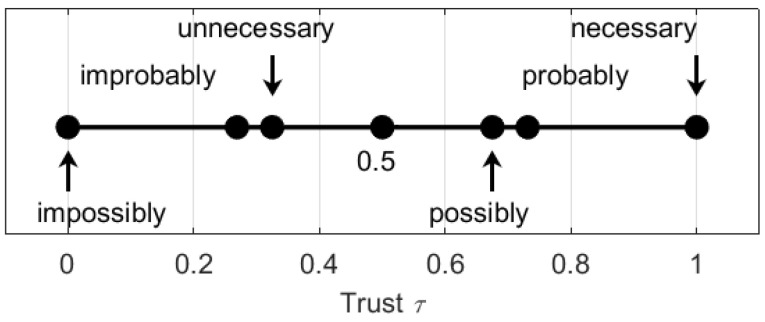
Interpretation of the trusts’ values.

The trusts defined by these functions for the pairs of necessity-impossibility, probability-improbability, and possibility-unnecessity are shown in Figure 2.

It is seen that on one side the trust τ(A)=0.73 in probable events is higher than the trust τ(A)=0.67 in possible events, but is lower than the trust τ(A)=1.00 in necessary events. On the other side, the trust τ(A)=0.27 in improbable events is higher than the trust τ(A)=0.00 in impossible events, but is lower than the trust τ(A)=0.33 in unnecessary events.

If A is interpreted as a proposition, then from a logical point of view the indicated truth values are interpreted as follows:

1. τ(A)=1 means that A is objectively true and τ(A)=0 means that A is objectively false;
2. $\tau \left(A\right)=w^{-1}\left(1\right)$ means that A is subjectively true (true from the observer’s point of view) and $\tau \left(A\right)=w^{-1}\left(-1\right)$ means that A is subjectively false (false from the observer’s point of view);
3. $\tau \left(A\right)=w^{-1}\left(w^{-1}\left(1\right)\right)$ means that A seems to be true and $\tau \left(A\right)=w^{-1}\left(-w^{-1}\left(1\right)\right)$ means that A is seems to be false.

The truth values of the subjective truth and false are denoted by $I_{w}$ and $O_{w}$, respectively, and these values hold the following true [16,18]:

(8) $$0<O_{w}<\eta <I_{w}<1,$$

where 0 and 1 represent Boolean false and true values that are limiting values for subjective false and subjective true, respectively. The truth value η indicates that it is undecidable whether the proposition A is true or false.

The suggested algebra with uninorm and absorbing norm operations allows direct definition of the control of mobile robots and their swarming. In the next section, we define such a system using the extension of the considered algebra based on the model of a neural network with mobile neurons.

### 2.2. Algebra of Control Variables

Let us adopt the presented above algebra A to direct the handling of the control variables. For convenience, assume that the control variables obtain their values from the interval [−1, 1]. Such an assumption is certainly not necessary, but it meets most cases of the control systems.

Formally it is required to extend algebra A to the interval [−1, 1]. The first option is to apply direct and inverse linear transformations

(9) c=2x−1,   x∈[0, 1],

(10) x=(c+1)/2,   c∈[−1, 1].

Then, the values c of control variables are converted to the trusts x and then after appropriate handling the resulting trusts x are converted to the controlled values c.

The second option is to define the extended algebra $A^{*}$, which inherits the properties of the algebra A, but acts on the interval [−1, 1]. Let $u^{*}:\left(-1,\;1\right)\to \left(-\infty ,\infty \right)$ and $v^{*}:\left(-1,\;1\right)\to \left(-\infty ,\infty \right)$ be continuous, strictly monotonously increasing functions with the zeroes $u^{*}\left(\theta^{*}\right)=v^{*}\left(\vartheta^{*}\right)=0$ and with the limits $\underset{x\to -1}{\lim}u^{*}\left(x\right)=\underset{x\to -1}{\lim}v^{*}\left(x\right)=-\infty$ and $\underset{x\to 1}{\lim}u^{*}\left(x\right)=\underset{x\to 1}{\lim}v^{*}\left(x\right)=+\infty$.

Using these functions, the extended uninorm ${⊕}_{\theta }^{*}:\left[-1,\;1\right]\times \left[-1,\;1\right]\to \left[-1,\;1\right]$ and absorbing norm ${⊗}_{\vartheta }^{*}:\left[-1,\;1\right]\times \left[-1,\;1\right]\to \left[-1,\;1\right]$ are defined similarly to the Equations (1) and (2):

(11) $$x{⊕}_{\theta }^{*}y=u^{*}{}^{-1}\left(u^{*}\left(x\right)+u^{*}\left(y\right)\right),$$

(12) $$x{⊗}_{\vartheta }^{*}y=v^{*}{}^{-1}\left(v^{*}{}^{-1}\left(x\right)\times v^{*}{}^{-1}\left(y\right)\right).$$

The inverse operations then are

(13) $$x{⊖}_{\theta }^{*}y=u^{*}{}^{-1}\left(u^{*}\left(x\right)-u^{*}\left(y\right)\right),$$

(14) $$x{⊘}_{\vartheta }^{*}y=v^{*}{}^{-1}\left(v^{*}\left(x\right)/v^{*}\left(y\right)\right),$$

where $v^{*}\left(y\right)\neq 0$.

Consequently, an extension of the algebra A to the interval [−1, 1] is a triple $A^{*}=\left(\left[-1,1\right],{⊕}_{\theta }^{*},{⊗}_{\vartheta }^{*}\right)$, in which extended uninorm ${⊕}_{\theta }^{*}$ and absorbing norm ${⊗}_{\vartheta }^{*}$ specify algebraic operations with zeroes $\theta^{*}$ and $\vartheta^{*}$, respectively.

Algebra $A^{*}$ inherits the properties of the algebra A, but since it is defined on the interval [−1,1] the trusts x∈[−1,1] can be directly considered as control variables c and vice versa. This algebra can be considered as a continuous version of both the balanced ternary numerical system and the ternary logic.

The defined algebra $A^{*}$ can be used for direct definition of the control and learning system. Below we consider the extension of previously defined neural network with mobile neurons. This network is a basis for specification of navigation and swarming of mobile robots.

## 3. Neural Network with Mobile Neurons in Algebra $A^{*}$

Control of the mobile robots is defined using the extended algebra presented above and uninorm and absorbing norm aggregators. Such an approach allows direct application of formal operations for specification of the robots’ actions. The swarming process is then defined using the model of neural network with mobile Tsetlin neurons.

### 3.1. States of the Neurons and of the Synapses

In general, in a neural network with mobile neurons it is assumed that the connectivity between the neurons depends on the distance between the neurons, and then the training process leads to restructuring the network and forming ensembles of the neurons. On the other hand, the distance between the neurons is defined with respect to the connectivity between the neurons; thus, the learning process is governed by the neurons motion [18].

Let us consider a neural network defined in the algebra $A^{*}$. Formally this network follows the previously defined Tsetlin network acting in algebra A [13], but the use of the interval [−1, 1] instead of the interval [0, 1] results in more convenient specification of the neurons’ motion.

Assume that the ith neuron obtains the inputs via l synapses and that at each time t=0,1,2,… the input value $x_{k}\left(t\right)$ appears at the kth synapse, k=1,2,…,l, which is characterized by the weight $\omega^{k}\left(t\right)$. The weight can be considered as a transition possibility, which is the ability of the synapse to transfer the input value or as a transition trust that is the trust that the synapse transmits the input value. In the considered framework, transition possibility and transition trust have the same meaning and specify the level of connectivity between the neurons. At the same time, as a logical value, the weight $\omega^{k}\left(t\right)$ is an operand of multi-valued logical operations.

In the Tsetlin network defined in $A^{*}$, each neuron acts as follows. The input value $x_{k}\left(t\right)$ is aggregated with the weight $\omega^{k}\left(t\right)$ by the uninorm ${⊕}_{\theta }^{*}$ or by the absorbing norm ${⊗}_{\vartheta }^{*}$ and the resulting value is transmitted to the neuron. The neuron compares the obtained value with its internal state $s_{i}\left(t\right)$ using the absorbing norm ${⊗}_{\vartheta }^{*}$. Finally, using the uninorm ${⊕}_{\theta }^{*}$ the neuron aggregates the results of such a comparison for all l inputs and specifies the obtained result as an output $z_{i}\left(t\right)$, which can also be (or can be not) used as the next internal state $s_{i}\left(t+1\right)$.

Formally, such activity is defined as follows. Let $\left(x_{1}\left(t\right),x_{2}\left(t\right),\ldots ,x_{l}\left(t\right)\right)$ be the values appearing at the synapses of the neuron at time t, $s_{i}\left(t\right)$ be the neuron’s internal state and $\left(\omega^{1}\left(t\right),\omega^{2}\left(t\right),\ldots ,\omega^{l}\left(t\right)\right)$ be the weights of the neuron’s synapses. The output values of synapses k=1,2,…,l are defined using the uninorm ${⊕}_{\theta }^{*}$ (*u*-synapses)

(15) $$y_{k}\left(t\right)=x_{k}\left(t\right){⊕}_{\theta }^{*}\omega^{k}\left(t\right)$$

or using the absorbing norm ${⊗}_{\vartheta }^{*}$ (*a*-synapses)

(16) $$y_{k}\left(t\right)=x_{k}\left(t\right){⊗}_{\vartheta }^{*}\omega^{k}\left(t\right).$$

Then each of these values is compared with the current state of the neuron using the absorbing norm, that is

(17) $$c_{k}\left(t\right)=y_{k}\left(t\right){⊗}_{\vartheta }^{*}s_{i}\left(t\right),$$

and, finally, the results of comparisons are aggregated using the uninorm, and the output of the neuron is specified by

(18) $$z_{i}\left(t\right)={⊕}_{\theta }^{*}{}_{k=1}^{l}c_{k}\left(t\right).$$

The next state $s_{i}\left(t+1\right)$ of the neuron is specified by a certain state function, which in the simplest case is

(19) $$s_{i}\left(t+1\right)=z_{i}\left(t\right).$$

Let us consider the weights ω(t)∈[−1,1] of the synapses, which specify the strength of the connection between the neurons. The changes of these values are associated with the learning process. The weights can be defined either externally by a certain training process or internally using the states of the neurons. In the first case, the network converges to definite configuration, which leads to the desired result on the output neurons, while in the second case the network demonstrates certain self-organization [27] and can change its configuration with respect to the states of the neurons and of the environment. In the suggested model, we follow the second approach and define the transition possibilities with respect to the states of the interconnecting neurons.

Let $N_{i}$ and $N_{j}$ be two neurons connected by the synapse $S_{ij}$. The states of the neurons at time t are denoted by $s_{i}\left(t\right)$ and $s_{j}\left(t\right)$ and the weight of the synapse is denoted by $\omega_{ij}\left(t\right)$. Notice that in the notation of the weight $\omega^{k}$ the upper index stands for kth input of the neuron and in the notation $\omega_{ij}$ the bottom pair of indices represents the neurons interconnected by the considered synapse. Using the absorbing norm ${⊗}_{\vartheta }^{*}$, the value $\omega_{ij}\left(t\right)$ is defined as

(20) $$\omega_{ij}\left(t\right)=s_{i}\left(t\right){⊗}_{\vartheta }^{*}s_{j}\left(t\right),$$

which, in essence, is a result of the comparison between the neurons’ states $s_{i}\left(t\right)$ and $s_{j}\left(t\right)$.

**Figure 3:**
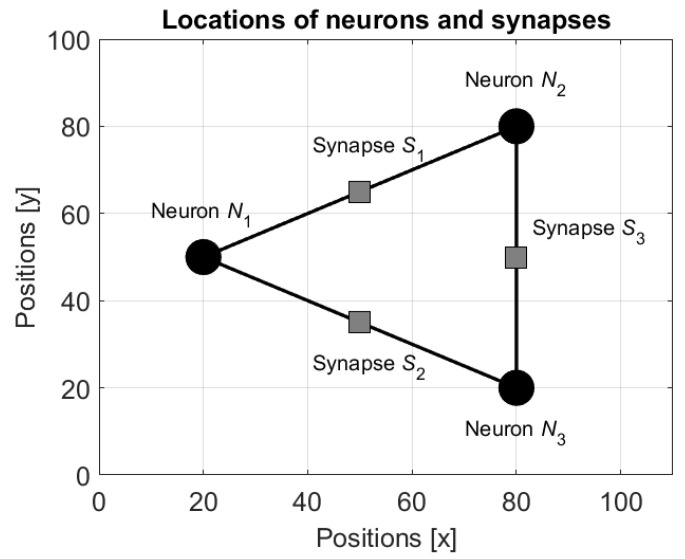
Locations of three neurons $N_{1}$, $N_{2},$ and $N_{3}$ and three synapses $S_{1}$, $S_{2},$ and $S_{3}$ connecting the neurons.

In the network with mobile neurons, the weights $\omega_{ij}\left(t\right)$ are applied for two goals. The first is a conventional use for specification of the output $y_{j}\left(t\right)$ of the neuron $N_{j}$ (see Equations (15) and (16)), and the second is a definition of the value of the potential function u in the location of the synapse. The second use is possible since in the considered approach the network is implemented in the form of physical devices with definite locations on a plane. Namely, if the neurons $N_{i}$ and $N_{j}$ are associated with the mobile robots located in the points $c_{i}$ and $c_{j}$, then it is assumed that the synapse is located at the geometrical center ${\bar{c}}_{ij}$ of the line connecting these points. Figure 3 shows an example of locations of three neurons $N_{1}$, $N_{2}$, and $N_{3}$ and three synapses $S_{1}$, $S_{2}$, and $S_{3}$ connecting these neurons.

**Figure 4:**
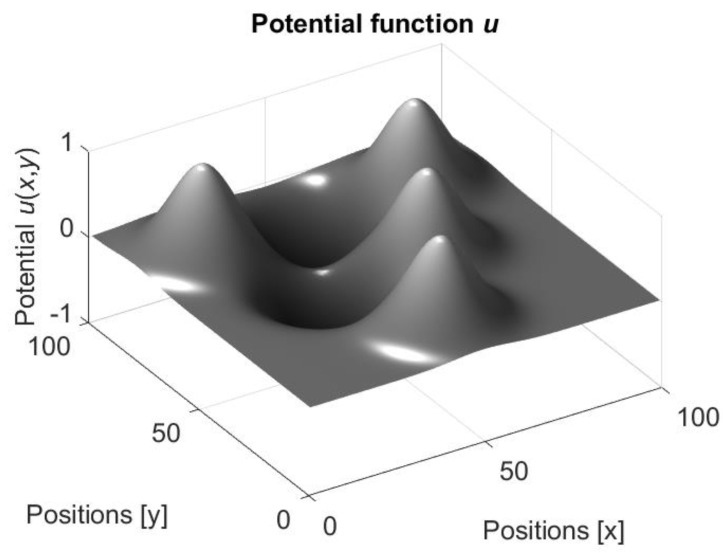
Potential function for the neurons and the synapses shown in Figure 3. The states of the neurons are $s_{1}=-1$, $s_{2}=1$ and $s_{3}=1$ and the weights of the connections (the states of the synapses) are $\omega_{13}=\omega_{12}=-1$ and $\omega_{23}=1$.

In the definition of the potential function, the neurons and, consequently, the robots are considered as obstacles and the potential function in their locations obtains the highest value 1. In the other points of the plane the values of the potential function are defined by an appropriate smoothing. The potential function for the neurons and the synapses shown in Figure 3 with the neurons’ states $s_{1}=-1$, $s_{2}=1$ and $s_{3}=1$ and the weights $\omega_{13}=\omega_{12}=-1$ and $\omega_{23}=1$ is shown in Figure 4.

It is seen that the locations of the neurons and the location of the third synapse that connects the neurons $N_{2}$ and $N_{3}$ potential are positive and the neurons (which are the mobile robots) are repulsed from these locations. However, the locations of the synapses that connect the neuron $N_{1}$ with the neurons $N_{2}$ and $N_{3}$ potential are negative, and the neurons (or robots) are attracted to these locations.

### 3.2. Reactive Learning and Motion of the Neurons

**Figure 5:**
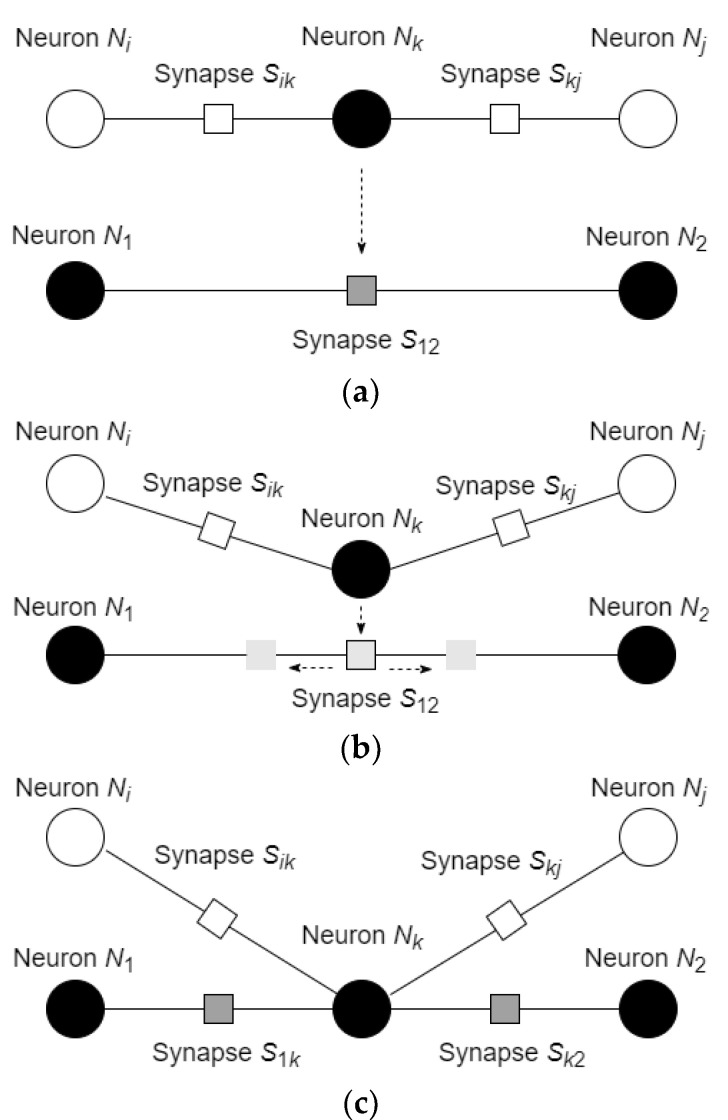
The process of synapse substitution and creation of new synapses: (**a**) neuron $N_{k}$, which is connected with the neurons $N_{i}$ and $N_{j}$ via the synapses $S_{ik}$ and $S_{kj}$, moves toward the synapse $S_{12}$, which connects the neurons $N_{1}$ and $N_{2}$; (**b**) the synapse $S_{12}$ is “divided” into two synapses; (**c**) neuron $N_{k}$ substitutes synapse $S_{12}$ in its location and connects with the neurons $N_{1}$ and $N_{2}$ via new synapses $S_{1k}$ and $S_{k2}$.

Learning in the considered network is based on the changes of the connectivity between the neurons and on the movements of the neurons with respect to the defined potential function. In addition, it is assumed that the neuron can substitute the synapse, to which location the neuron arrived, which results in creating two new synapses, connecting the arrived neuron with the neurons, which were connected by the substituted synapse. The process of synapse substitution and creation of new synapses is shown in Figure 5.

In the considered scenario, synapse $S_{12}$ before substitution had a negative value and attracted the neuron. After division, the synapse $S_{1k}$ starts with the values equivalent to the value of synapse $S_{12}$ and then changes it to the value calculated by Equation (20) with respect to the states of the neurons $N_{1}$ and $N_{k}$ (for synapse $S_{1k}$) and $N_{k}$ and $N_{2}$ (for synapse $S_{k2}$).

**Figure 6:**
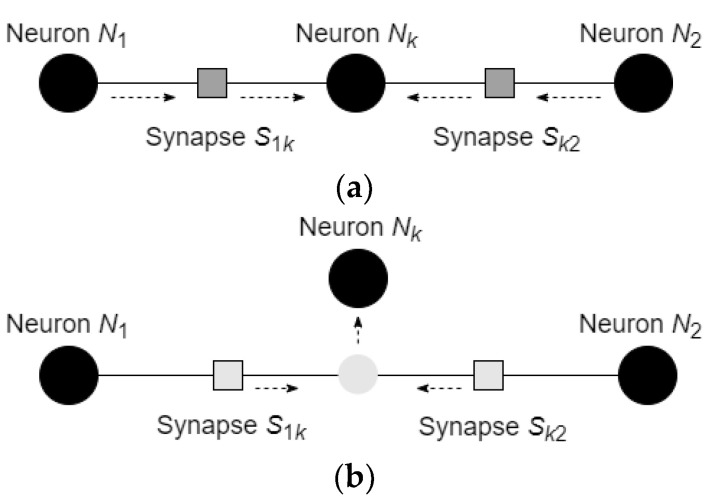

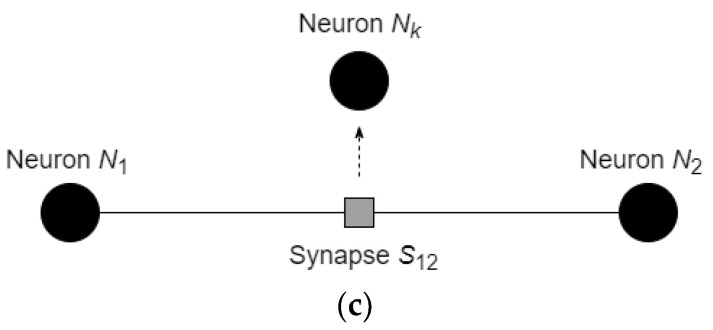
The process of repulsion of the neuron and union of the synapse: (**a**) neurons $N_{1}$ and $N_{2}$ repulse neuron $N_{k}$; (**b**) synapses $S_{1k}$ and $S_{k2}$ move toward each other; (**c**) synapses $S_{1k}$ and $S_{k2}$ are united into the synapse $S_{12}$.

In a similar manner, two neighboring neurons can repulse the neuron with the same sign of the state value. The process, in which the neurons $N_{1}$ and $N_{2}$ repulse neuron $N_{k}$, is shown in Figure 6.

Similar to the Coulomb law for the electrically charged particles, in the considered model it is assumed that each neuron attracts the neurons with different signs of the states and repulses the neurons with the same sign of the state. Using the value

(21) $$arp\left(N_{i},N_{j}\right)=u^{*}{}^{-1}\left({⊖}_{\theta }^{*}\left(s_{i}{⊗}_{\vartheta }^{*}s_{j}\right)\right)=u^{*}{}^{-1}\left({⊖}_{\theta }^{*}\omega_{ij}\right),$$

of attraction/repulsion between the neurons $N_{i}$ and $N_{j}$, where $s_{i}$ and $s_{j}$ are the states of the neurons $N_{i}$ and $N_{j}$, respectively, the attraction/repulsion force for these neurons is defined by the formula

(22) $$F\left(N_{i},N_{j}\right)=\lambda \;arp\left(N_{i},N_{j}\right)/dist\left(N_{i},N_{j}\right),$$

where $dist\left(N_{i},N_{j}\right)$ is a geometrical distance between the neurons and λ is an attraction/repulsion coefficient. In the Equation (21), the value $s_{i}{⊗}_{\vartheta }^{*}s_{j}$ represents similarity between the states, and in the Equation (22) the distance between the neurons is defined by the metric of the space, where the network acts; in the considered case it is Euclidian distance.

Certainly, attraction/repulsion can be defined differently with respect to the needs and requirements of the network. For example, attraction and repulsion can be defined using the known aggregation function [19]

(23) $$J_{agr}\left(N_{i},N_{j}\right)=-dist\left(N_{i},N_{j}\right)\left(a-b\exp\left(-\frac{dist^{2}\left(N_{i},N_{j}\right)}{c}\right)\right),$$

where the states of the synapses and of the neurons are expressed by the parameters a, b, and c, or by using different similarity measures.

### 3.3. Simulation of the Network Activity

To clarify activity of the defined network with mobile neurons let us consider the following example. Assume that the network is in the gridded square domain of the size N×N=100×100 cells and that the number of the neurons in the network is n=25.

Each neuron $N_{i}$, i=1,2,…,n, is connected with the neighboring neurons $N_{j}$, $j=1,2,\ldots n_{i}\leq n-1$, j≠i, which are located at the distances $dist\left(N_{i},N_{j}\right)$ bounded by certain predefined threshold $d_{max}$. For the illustrations below we used Euclidian distance and the threshold $d_{max}=30$.

If the distance $dist\left(N_{i},N_{j}\right)$ is less than the threshold $d_{max}$, then the state of the synapse $S_{ij}$ connecting the neurons $N_{i}$ and $N_{j}$, which is the weight $\omega_{ij}$, is calculated according to Equation (20) with respect to the current states $s_{i}\left(t\right)$ and $s_{j}\left(t\right)$ of the neurons $N_{i}$ and $N_{j}$, correspondingly. Otherwise, if the distance $dist\left(N_{i},N_{j}\right)$ is greater than the threshold $d_{max}$, then it is assumed that the neurons are not connected and the weight $\omega_{ij}$ is set to zero.

The attraction/repulsion force $F\left(N_{i},N_{j}\right)$ for the neurons $N_{i}$ and $N_{j}$ is calculated according to Equation (22), where attraction/repulsion coefficient λ is a tenths part of the domain diagonal, that is, $\lambda =0.1\sqrt{2N^{2}}=10\sqrt{2}=14.14$.

Following the attraction/repulsion forces, the neurons move in the resultant directions by the steps proportional to the values of the resultant attraction/repulsion forces. In the illustrations it is assumed that the lengths δ of the steps are equivalent to the values of the attraction/repulsion forces.

**Figure 7:**
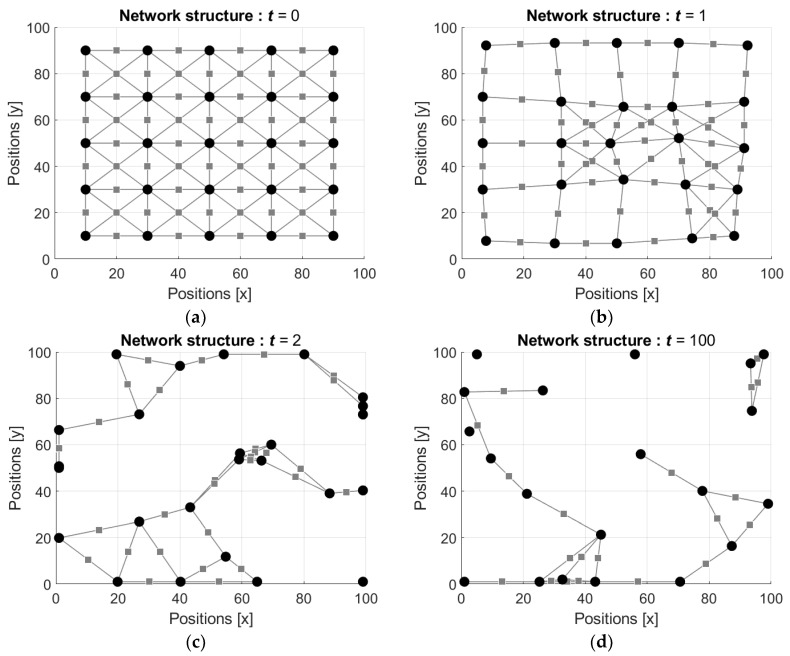
Evolution of the network structure: (**a**) initial regular configuration of the network, t=0; (**b**) configuration after the first movement of each neuron, t=1; (**c**) configuration after two movements of each neuron, t=2; and (**d**) configuration after hundred movements of each neuron, t=100.

**Figure 8:**
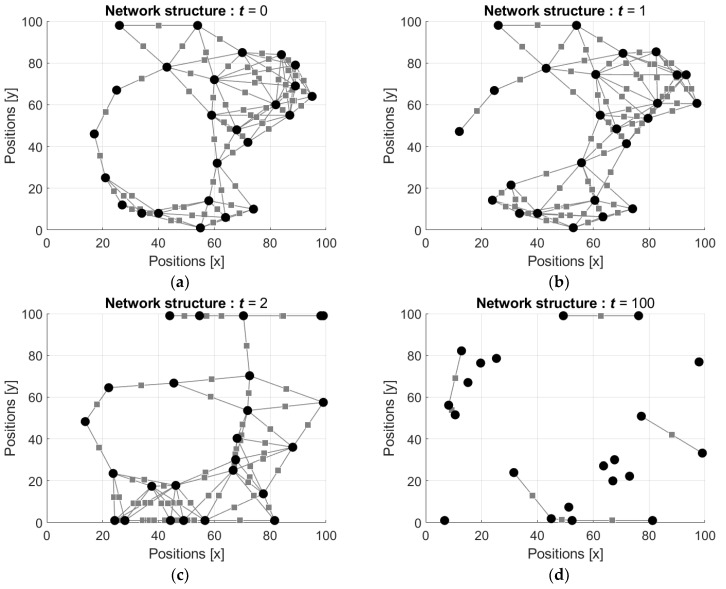
Evolution of the network structure: (**a**) initial random configuration of the network, t=0; (**b**) configuration after the first movement of each neuron, t=1; (**c**) configuration after two movements of each neuron, t=2; and (**d**) configuration after hundred movements of each neuron, t=100.

The states $s_{i}\left(t\right)$ of the neurons are initialized by random such that $s_{i}\left(0\right)$ are drawn from the interval [−1, 1] with respect to uniform distribution, and then these values as well as the weights of the connections are updated using the Equations (15)–(19). Examples of the evolution of the network structures are shown in Figure 7 and Figure 8 (the videos of the evolution of the network structures can be found in Supplementary Materials, Videos S1 and S2, respectively). In Figure 7 the starting configuration is a regular lattice and in Figure 8 the starting configuration is random.

It is seen that initially the regular configuration of the network (Figure 7a) is disturbed by the first movements of the neurons (Figure 7b), and this distortion is enough for serious change of the network configuration at the next step (Figure 7c). Figure 7d shows the neuron groups observed after the 100th step of each neuron.

Evolution of the network configuration starting from random configuration is shown in Figure 8.

It is seen that, similar to the previous example, the first motion of the neurons slightly changes the initial configuration of the network (cf. Figure 8a,b). However, already at the third step the configuration of the network changes seriously (Figure 8c). Figure 8d shows the configuration of the network after the 100th step of each neuron, which demonstrates that the initially connected network is divided to non-connected neurons, which preserve their locations. 

**Figure 9:**
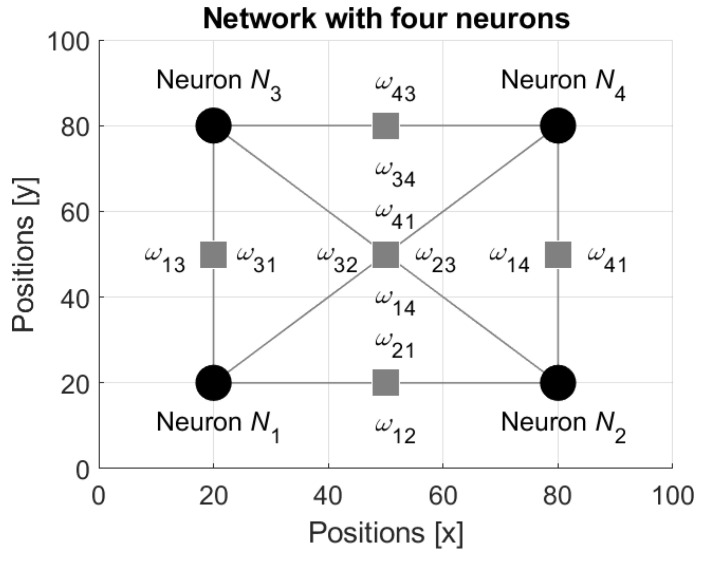
The network with four interconnected neurons.

In order to clarify the evolution of the network structure, let us consider the following example. Assume that the network includes n=4 interconnected neurons as it is shown in Figure 9.

Assume that the initial states $s_{i}\left(0\right)$ of the neurons $N_{i}$, i=1,…, 4, are

$$s_{1}\left(0\right)=-0.75,\;s_{2}\left(0\right)=-0.25,\;s_{3}\left(0\right)=0.25\;\mathrm{and}\;s_{4}\left(0\right)=0.75.$$

Then the weights $\omega_{ij}\left(0\right)$, i,j=1,…, 4, of the connections defined by Equation (20) are defined by the matrix:

$$\omega \left(0\right)=\left(\begin{array}{cc} \begin{array}{cc} 0 & 0.2435\\ 0.2435 & 0 \end{array} & \begin{array}{cc} -0.2435 & -0.7383\\ -0.0651 & -0.2435 \end{array}\\ \begin{array}{cc} -0.2435 & -0.0652\\ -0.7383 & -0.2435 \end{array} & \begin{array}{cc} 0 & 0.2435\\ 0.2435 & 0 \end{array} \end{array}\right).$$

Direct application of the Equations (15) and (17)–(19) results in the following states $s_{i}\left(1\right)$, i=1,…, 4, at the time t=1:

$$s_{1}\left(1\right)=-0.0256,\;s_{2}\left(1\right)=-0.0485,\;s_{3}\left(1\right)=-0.0817\;\mathrm{and}\;s_{4}\left(1\right)=-0.9534.$$

Then, the weights $\omega_{ij}\left(1\right)$, i,j=1,…, 4, are:

$$\omega \left(1\right)=\left(\begin{array}{cc} \begin{array}{cc} 0 & 0.0012\\ 0.0012 & 0 \end{array} & \begin{array}{cc} 0.0021 & 0.0478\\ 0.0040 & 0.0905 \end{array}\\ \begin{array}{cc} 0.0021 & 0.0040\\ 0.0478 & 0.0905 \end{array} & \begin{array}{cc} 0 & 0.1518\\ 0.1518 & 0 \end{array} \end{array}\right).$$

By the same manner, the states $s_{i}\left(1\right)$, i=1,…, 4, at the time t=2 are

$$s_{1}\left(2\right)=0.0498,\;s_{2}\left(2\right)=-0.0910,\;s_{3}\left(2\right)=-0.1450\;\mathrm{and}\;s_{4}\left(2\right)=-0.2477.$$

The weights $\omega_{ij}\left(2\right)$, i,j=1,…, 4, at this time are

$$\omega \left(2\right)=\left(\begin{array}{cc} \begin{array}{cc} 0 & 0.0046\\ 0.0046 & 0 \end{array} & \begin{array}{cc} 0.0073 & -0.0126\\ 0.0133 & -0.0231 \end{array}\\ \begin{array}{cc} 0.0073 & 0.0133\\ -0.0126 & -0.0231 \end{array} & \begin{array}{cc} 0 & -0.0369\\ -0.0369 & 0 \end{array} \end{array}\right).$$

Finally, already at the time t=3 the states become closer to zero

$$s_{1}\left(3\right)=-0.0008,\;s_{2}\left(3\right)=-0.0057,\;s_{3}\left(3\right)=-0.0187\;\mathrm{and}\;s_{4}\left(3\right)=-0.0542,$$

and the weights obtain the zero values $\omega_{ij}\left(3\right)=0$, i,j=1,…, 4,

ω(3)=(0).

Thus, the neurons are separate and at the next step, t=4, the states also obtain zero values

$$s_{1}\left(4\right)=0,\;s_{2}\left(4\right)=0,\;s_{3}\left(4\right)=0\;\mathrm{and}\;s_{4}\left(4\right)=0,$$

which are preserved infinitely. Since the movements of the neurons are defined by the similarity between the neurons’ states (see Equation (21)), then the motions are zeroed, and the neurons stay at their locations.

The dynamics of the neural network with mobile neurons allows both its usage in traditional machine learning applications, and as a model of swarm activity. In the next section we implement the second possibility and apply the constructed network for specification of the swarm activity.

## 4. Robot States and Movements

In the suggested approach, activity of the group of mobile robots is modeled using a neural network with mobile neurons. The states of the robots are associated with the states of the neurons, and the neurons’ assembling is associated with the robots’ swarming and creating subgroups in the swarm.

Motion of the neurons can be mapped into the motion of the robots in two ways: either by direct transformation of the neuron’s movements into the movements of the robot or by the use of separate networks for specification of the movements in different dimension. In the first case, the activity if the swarm mimics the activity of the network presented in Section 3.3, while in the second case the swarm dynamics demonstrate additional properties; below we consider the second definition of the swarm activity.

### 4.1. Robot States under Uncertainty

Consider a mobile robot acting on a plane and assume that the state of the robot is defined by its location and heading. The robot can move one step forward, turn left or right and then move one step forward in a new direction and stay in its current location with current heading.

If the state of the robot is certain, then the next state of the robot is defined by the chosen movement: if the movement is certain, then the next state is also certain and if the movement is uncertain, the next state is uncertain with the same grade of uncertainty. If the state (either the robot’s location or heading or both) is uncertain, then the next state of the robot is also uncertain and for a uncertain movement it includes both uncertainty of the state and of the movement.

In the probabilistic terms, uncertainties of the states and movements are defined by the states’ and transitions’ probabilities. Then the dynamics of the are is governed by the Markov process, which specifies the probabilities of the next robot’s locations and heading. In the terms of subjective trusts, the robot’s dynamics is defined by similar manner, but instead of the Markov process the subjective Markov process [13] is used.

Denote by $\xi \left(t\right)=\left(\xi_{1}\left(t\right),\xi_{2}\left(t\right)\right)$ the vector of the decision-maker’s trusts regarding the robot’s heading at time t. It is assumed that

- The trust vector ξ(t)=(1, 1) means that the heading of the robot is necessary “↑”;
- The trust vector ξ(t)=(−1,−1) means that the heading of the robot is necessary “↓”;
- The trust vector ξ(t)=(−1, 1) means that the heading of the robot is necessary “←”;
- The trust vector ξ(t)=(1,−1) means that the heading of the robot is necessary “→”.

The intermediate values $\xi_{1}\left(t\right),\xi_{2}\left(t\right)\in \left(-1,\;1\right)$ specify the levels of the trust that the robot has certain direction.

The trusts of the decision-maker about the turns of the robot are represented by the matrix

(24) $$T\left(t\right)=\|\begin{array}{cc} \tau_{11}\left(t\right) & \tau_{12}\left(t\right)\\ \tau_{21}\left(t\right) & \tau_{22}\left(t\right) \end{array}\|,$$

of the trusts such that

- The trust matrix $T\left(t\right)=\|\begin{array}{cc} 1 & 0\\ 0 & 1 \end{array}\|$ means preserving current direction of the robot with necessity;
- The trust matrix $T\left(t\right)=\|\begin{array}{cc} -1 & 0\\ 0 & 1 \end{array}\|$ means turn left with necessity;
- The trust matrix $T\left(t\right)=\|\begin{array}{cc} 1 & 0\\ 0 & -1 \end{array}\|$ means turn right with necessity.

The intermediate values $\tau_{ij}\left(t\right)\in \left(-1,\;1\right)$, i,j=1,2, specify the levels of the trust that the robot turns in certain direction.

Notice that the suggested notation coincides (up to inambiguous mapping) with the usual definition of headings by the vectors ξ=(0, 1)≜“↑”, ξ=(0,−1)≜“↓”, ξ=(−1, 0)≜“←” and ξ=(1, 0)≜“→”, and of the turns by the rotation matrix

(25) $$R=\|\begin{array}{cc} \cos\left(\varphi \right) & \sin\left(\varphi \right)\\ -\sin\left(\varphi \right) & \cos\left(\varphi \right) \end{array}\|,$$

where φ is a rotation angle such that $R=\|\begin{array}{cc} 1 & 0\\ 0 & 1 \end{array}\|$ means “preserve the current heading”, $R=\|\begin{array}{cc} 0 & 1\\ -1 & 0 \end{array}\|$ means “turn left” and $R=\|\begin{array}{cc} 0 & -1\\ 1 & 0 \end{array}\|$ means “turn right”. In addition, the trust vector ξ(t) and the matrix T(t) can be considered in the terms of quantum controlled mobile robots and the observations of their states [28] that provides an additional framework for the suggested model.

The turns of the robot in the terms of the trusts are defined by the same manner as its turns using rotation matrix. Namely, if $\xi \left(t\right)=\left(\xi_{1}\left(t\right),\xi_{2}\left(t\right)\right)$ is the trust vector of the robot’s state at time t, then the trust vector $\xi \left(t+1\right)=\left(\xi_{1}\left(t+1\right),\xi_{2}\left(t+1\right)\right)$ of the state at time t+1 is defined as follows

(26) $$\xi \left(t+1\right)=\xi \left(t\right){⊗}_{\vartheta }^{*}T\left(t\right),$$

where, similar to the usual multiplication rule of the vector and the matrix,

(27) $$\xi_{1}\left(t+1\right)=\left(\xi_{1}\left(t\right){⊗}_{\vartheta }^{*}\tau_{11}\left(t\right)\right){⊕}_{\theta }^{*}\left(\xi_{2}\left(t\right){⊗}_{\vartheta }^{*}\tau_{21}\left(t\right)\right),\;\xi_{2}\left(t+1\right)=\left(\xi_{1}\left(t\right){⊗}_{\vartheta }^{*}\tau_{12}\left(t\right)\right){⊕}_{\theta }^{*}\left(\xi_{2}\left(t\right){⊗}_{\vartheta }^{*}\tau_{22}\left(t\right)\right).$$

Evolution of the trusts defined by Equation (26) is known as the subjective Markov process [13].

In order to define transition trusts $\tau_{ij}\left(t\right)$, i,j=1,2, let us associate the values $\xi_{1}\left(t\right)$ and $\xi_{2}\left(t\right)$ as the states $s_{1}\left(t\right)$ and $s_{2}\left(t\right)$ of the Tsetlin neurons $N_{1}$ and $N_{2}$ (see Section 3.1), which are connected with themselves and with each other. Then, following the definitions of the neuron’s functions (15)–(19), the transition trusts are defined as

(28) $$\tau_{11}\left(t\right)={⊕}_{\theta }^{*}{}_{k=1}^{l}\left(x_{k}\left(t\right){⊕}_{\theta }^{*}\omega_{k1}\left(t\right)\right),\;\tau_{12}\left(t\right)=x_{1}\left(t\right){⊕}_{\theta }^{*}\omega_{12}\left(t\right),\;\tau_{21}\left(t\right)=x_{2}\left(t\right){⊕}_{\theta }^{*}\omega_{21}\left(t\right),\;\tau_{22}\left(t\right)={⊕}_{\theta }^{*}{}_{k=1}^{l}\left(x_{k}\left(t\right){⊕}_{\theta }^{*}\omega_{k2}\left(t\right)\right),$$

or as

(29) $$\tau_{11}\left(t\right)={⊕}_{\theta }^{*}{}_{k=1}^{l}\left(x_{k}\left(t\right){⊗}_{\vartheta }^{*}\omega_{k1}\left(t\right)\right),\;\tau_{12}\left(t\right)=x_{1}\left(t\right){⊗}_{\vartheta }^{*}\omega_{12}\left(t\right),\;\tau_{21}\left(t\right)=x_{2}\left(t\right){⊗}_{\vartheta }^{*}\omega_{21}\left(t\right),\;\tau_{22}\left(t\right)={⊕}_{\theta }^{*}{}_{k=1}^{l}\left(x_{k}\left(t\right){⊗}_{\vartheta }^{*}\omega_{k2}\left(t\right)\right),$$

with respect to the type of synapses—*u*-synapses (Equation (15)) or *a*-synapses (Equation (16))—used in the connections between the neurons. 

**Figure 10:**
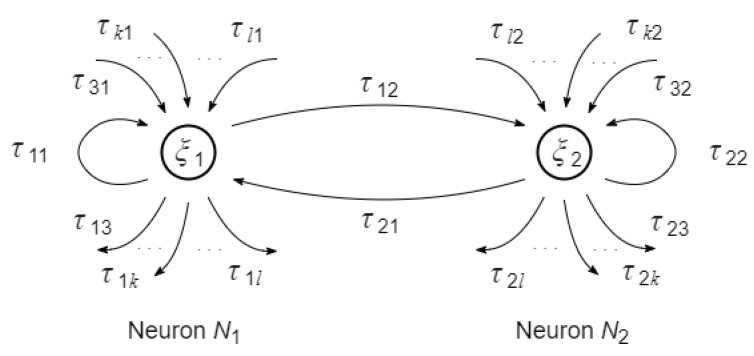
Connection of the neurons that represent the state of the robot.

Connections of the neurons are shown in Figure 10.

In the figure, the transition trusts $\tau_{ij}\left(t\right)$, i,j=1,2, represent internal controls in the robot and the trusts $\tau_{ij}\left(t\right)$, i,j=3,…,k,…l, represent connections of the robot with the other robots in the swarm.

Finally, let us consider the mobility of the neurons and its use for definition of the robots’ swarming. Let ${ℜ}_{1}$ and ${ℜ}_{2}$ be two mobile robots moving on a plane by conducting the indicated above motions.

Assume that the state of each robot is defined by the corresponding trust vector: vector $\xi^{1}\left(t\right)=\left(\xi_{1}^{1}\left(t\right),\xi_{2}^{1}\left(t\right)\right)$ for the robot ${ℜ}_{1}$ and vector $\xi^{2}\left(t\right)=\left(\xi_{1}^{2}\left(t\right),\xi_{2}^{2}\left(t\right)\right)$ for the robot ${ℜ}_{2}$. As above, let us associate the elements of the trust vectors $\xi^{1}\left(t\right)$ and $\xi^{2}\left(t\right)$ with the states of the neurons. In other words, we assume that the state of each robot is defined by the pair of neurons, that is ${ℜ}_{1}=\left(N_{1}^{1},N_{2}^{1}\right)$ and ${ℜ}_{2}=\left(N_{1}^{2},N_{2}^{2}\right)$, and the states $s_{i}^{j}\left(t\right)$ of the neurons are associated with the trusts $\xi_{i}^{j}\left(t\right)$, i,j=1,2.

Then, the attraction and repulsion between the robots are the combination of the attraction and repulsion between each pair of the neurons associated with the robots. Using Equation (22), the attraction/repulsion force $F\left({ℜ}_{1},{ℜ}_{2}\right)$ between the robots consists of four forces

(30) $$F\left({ℜ}_{1},{ℜ}_{2}\right)=\left\{F\left(N_{i}^{1},N_{j}^{2}\right)\;:i,j=1,\;2\right\}.$$

**Figure 11:**
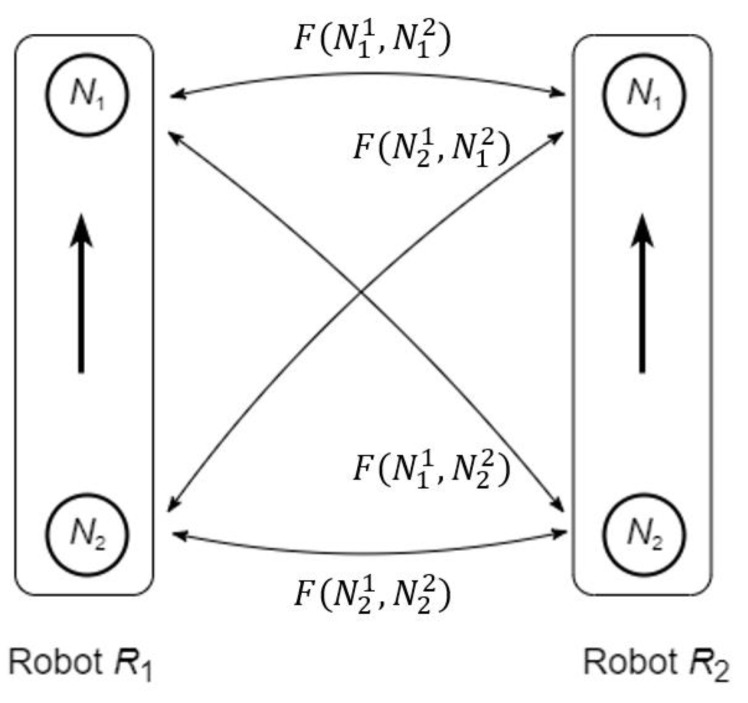
Attraction/repulsion forces between the robots. The bold arrows indicate the heading of the robots.

The scheme of attraction and repulsion of the robots is shown in Figure 11.

It is seen that each robot together with the applied forces can be considered as a dipole. Consequently, the group of the robots forms a dipole dynamical system [24] governed by the rules based on extended uninorm and absorbing norm. Below, we present numerical simulations of the behavior of such system.

### 4.2. Simulation of the Robots’ Motion and Swarming

Activity of the group of the robots was simulated in a similar setting as the above considered activity of the neural network with mobile neurons. The group includes n=25 robots ${ℜ}_{i}=\left(N_{1}^{i},N_{2}^{i}\right)$, i=1,2,…,n, acting in the gridded square domain of the size N×N=100×100.

Each neuron $N_{1}^{i}$ and $N_{2}^{i}$ of each robot ${ℜ}_{i}$ is connected with the neurons $N_{1}^{j}$ and $N_{2}^{j}$ of the neighboring robot ${ℜ}_{j}$, $j=1,2,\ldots ,n_{i}\leq n-1$, j≠i, which are located at the distances less or equal to the threshold $d_{max}$. As above, in the simulations the distances are Euclidian, and the threshold is $d_{max}=30$. The value of the attraction/repulsion coefficient λ is also $\lambda =0.1\sqrt{2N^{2}}=14.14$.

**Figure 12:**
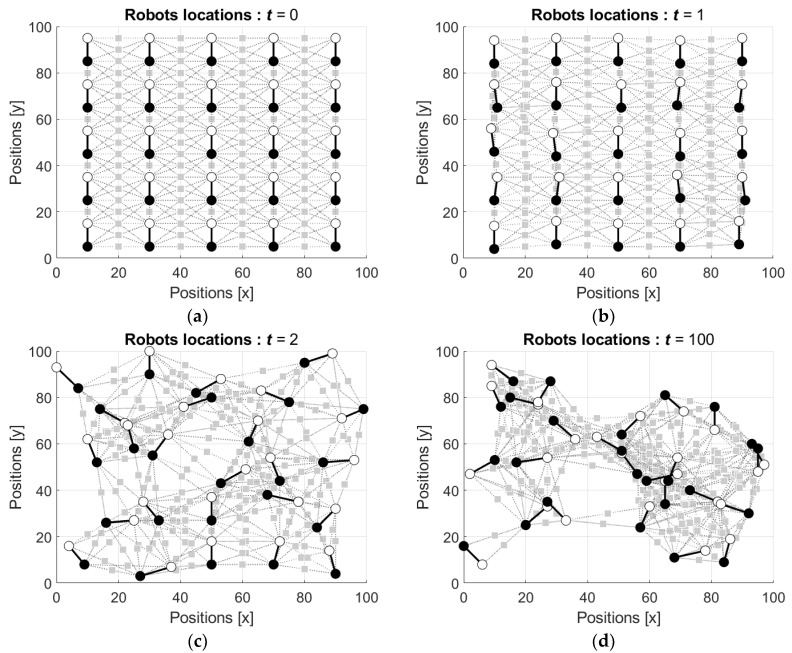
Locations of the robots starting from the position in ordered configuration: (**a**) initial locations, t=0; (**b**) locations after the first movement of each robot, t=1; (**c**) locations after the second movement of each robot, t=2; and (**d**) locations after the 100th movement of each robot, t=100.

In the first simulations, the states $s_{1}^{i}\left(t\right)$ and $s_{2}^{i}\left(t\right)$ of the neurons are initialized by random, such that both $s_{1}^{i}\left(t\right)$ and $s_{2}^{i}\left(t\right)$ are drawn from the interval [−1 1] with respect to uniform distribution and then these values as well as the weights of the connections are updated using Equations (15)–(19). Figure 12 shows the locations of the robots starting from the ordered configuration (the video can be found in Supplementary Materials, Video S3). In this figure and the figures below the neuron $N_{1}^{i}$ is depicted by a white circle and the neuron $N_{2}^{i}$ is depicted by a black circle.

It is seen that similar to the behavior of the neural network with mobile robots (see Figure 7), the initially ordered configuration of the robots’ locations (Figure 12a) is disturbed by the first movements of the robots (Figure 12b), and this distortion increases with the next movement (Figure 12c). Figure 12d shows configuration of the robots’ locations after the 100th movement of each robot.

**Figure 13:**
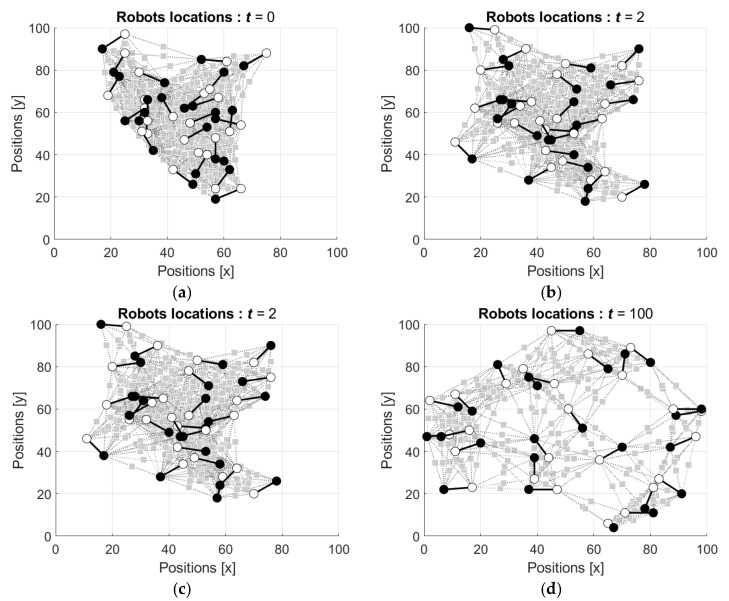
Locations of the robots starting from the position in random configuration: (**a**) initial locations, t=0; (**b**) locations after the first movement of each robot, t=1; (**c**) locations after the second movement of each robot, t=2; and (**d**) locations after the 100th movement of each robot, t=100.

Locations of the robots starting from random configuration are shown in Figure 13 (the video can be found in Supplementary Materials, Video S4).

It is seen that the initially dense group of the robots (Figure 13a) diffuses by the first and the second movements of the robots (Figure 13b,c). Figure 13d shows scattered configuration of the robots’ locations after the 100th movement of each robot.

Notice that in both scenarios the states of both $N_{1}^{i}$ and $N_{2}^{i}$ in each ith robot, i=1,2,…,25, were initialized equivalently by random values from the interval [−1, 1]. In other words, the robots were not directed, and this fact is seen in the figures, where the neurons $N_{1}^{i}$ and $N_{2}^{i}$ attracted and repulsed with no concern to their index.

In the final simulations, in contrast, the states $s_{1}^{i}\left(t\right)$ and $s_{2}^{i}\left(t\right)$ of the neurons are initialized by random, such that $s_{1}^{i}\left(0\right)$ are drawn from the interval [0, 1] and $s_{2}^{i}\left(t\right)$ are drawn from the interval [−1, 0] with respect to uniform distribution. Thus, in these scenarios the robots are directed in such a manner that the neurons with the states of opposite signs attract and the neurons with the states of the same signs repulse. The states and the weights of the connections are updated using the Equations (15)–(19).

**Figure 14:**
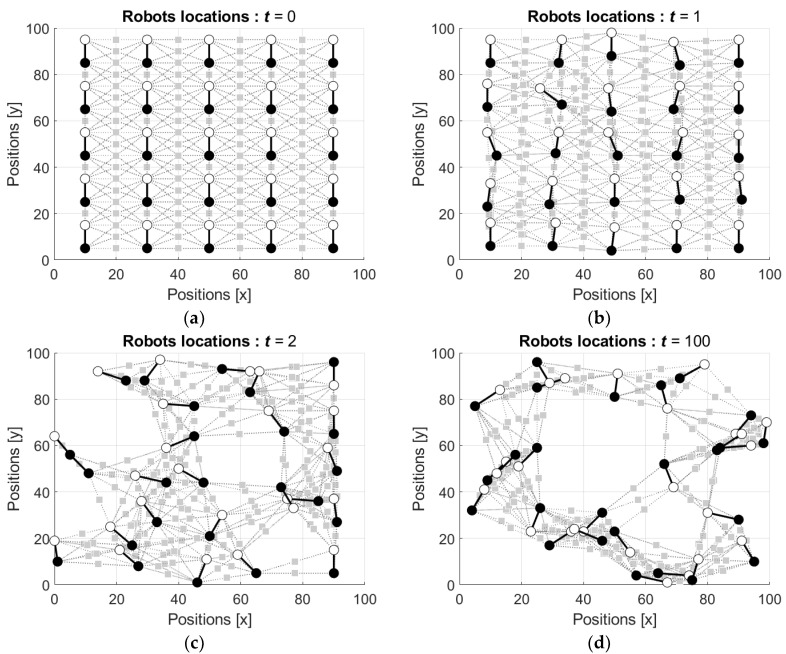
Locations of the directed robots starting from the position in ordered configuration: (**a**) initial locations, t=0; (**b**) locations after the first movement of each robot, t=1; (**c**) locations after the second movement of each robot, t=2; and (**d**) locations after the 100th movement of each robot, t=100.

As above, in the simulations the robots started from the locations in ordered configuration and from the locations in random configuration. Figure 14 shows the locations of the robots starting from the ordered configuration (the video can be found in Supplementary Materials, Video S5).

It is seen that the movement of the robots is similar to the movement in the scenario illustrated by Figure 12, with the expected difference in attraction and repulsion of the robots’ neurons with equal and different signs depicted by black and white circles, respectively.

**Figure 15:**
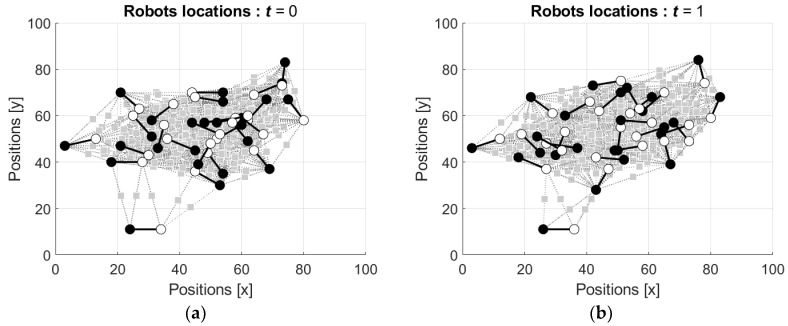

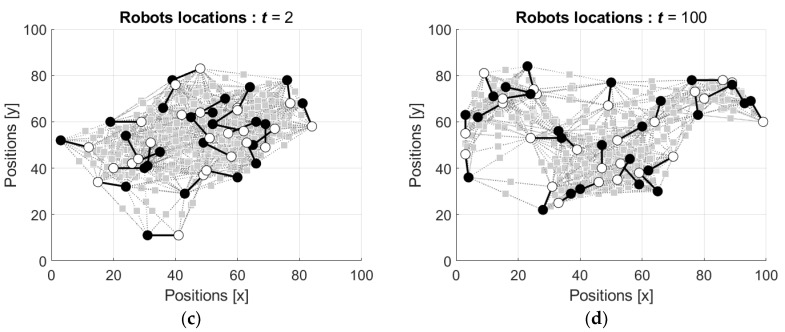
Locations of the directed robots starting from the position in random configuration: (**a**) initial locations, t=0; (**b**) locations after the first movement of each robot, t=1; (**c**) locations after the second movement of each robot, t=2; and (**d**) locations after the 100th movement of each robot, t=100.

Finally, locations of the robots starting from random configuration are shown in Figure 15 (the video can be found in Supplementary Materials, Video S6).

As it was expected, in this scenario, the motion of the robots is similar to the motion illustrated by Figure 13, with the already-mentioned difference in the repulsion because of the difference between the signs of the neurons’ states.

The simulations demonstrate that starting from arbitrary locations the robots demonstrate variative motion in the group. The states of the neurons in the robots differ from the boundary values; as a result, the connections between the robots are preserved. Together with that, the states and connections of some of the robots converge to the steady state values such that the robots keep their locations.

The observed simulated behavior of the robots follows the behavior of the suggested neural network with mobile neurons, which governs each of the robots’ neurons, which allows consideration of decision making and the actions in the same framework.

## 5. Discussion

The suggested approach of the control of mobile robots considers decision making and operating as a unified process based on the novel method of handling imperfect information and acting under uncertainty. The method is based on the extension of recently developed algebra with parameterized unary and binary aggregators, which implement multi-valued logical operations.

Formally, the suggested method follows thee general scheme of fuzzy control (see, e.g., [29,30]), but, in contrast to the usual techniques, it is focused on the algebraic model and on the relation between possibilities, probabilities, and necessities. The measures of uncertainty are considered as subjective trusts, which specify both decisions and actions of the system.

Control of the robots’ motion in the group is based on the neural network with the mobile neurons. The idea of such a network with mobile neurons was suggested by Apolloni et al. [18]. In addition to the usual activity of neural network, in this network the mobility of the neurons is applied, and it both defines and is defined by the connectivity between the neurons. In the suggested model the network consists of the Tsetlin neurons, and connections between the neurons and their mobility are governed by the states of the neurons. The activity of the network is defined in the developed algebra of the subjective trusts.

From a control point of view, each robot is specified as a dipole [24], which originally was used by Tchieu et al. in the model of behavior of fish flocks. In the suggested model, the robot consists of two mobile neurons acting in the neural network with obvious limitation led by the size of the robot. Such a model allows to consider the control of the robot by the same techniques as the control of quantum controlled mobile robots [6,18], but with stressing the difference in the topology of the actions’ space.

Since the suggested model applies subjective measures of uncertainty, control is considered from the internal point of view and follows the topology of a non-oriented surface. Such topology most likely differs from the topology of the controls used in the performance centered model by Saeidi [22,23] and of the controls in the model of quantum control [28,31]; these issues will be addressed in future studies.

Finally, the paper does not address teleologic issues or specific applications and can be considered as a proof of concept. Numerical simulations have demonstrated the feasibility of the suggested method. At the next steps, the suggested method is planned to be implemented in the search and foraging algorithms. In the simple case of following toward a single target, the exact values of the trusts can be defined using the recently calculated probabilities of altruistic and egoistic steps of the agents [11], while in more complicated scenarios, additional efforts and comparisons of the suggested method with the known techniques will be required.

## Supplementary Materials

The following supporting information can be downloaded at: https://www.mdpi.com/article/10.3390/e24060790/s1, Videos S1–S6.
